# Turning over New Leaves

**Published:** 1890-02-08

**Authors:** 


### Turning Over New Leaves.

**"Father Damien,"** *

By Edward Clifford, is a short description of a journey from Cashmere to the beautiful leper settlement at Molokai, where Father Damien de Veuster found his vocation, and whence last year he passed to his reward. The introductory chapter contains the author's reasons against joining the Church of Rome, as to the soundness of which every man must judge for himself. For the rest Mr. Clifford's style is manly and straightforward, his descriptions are genuine bits of word painting, and his story is plainly told. From the very nature of its subject Mr. Clifford's book is full of pathos, and an additional touch of this is imparted by the simplicity of diction with which it is written. The book concludes with a report of the first meeting of the Father Damien Memorial Fund at Marlborough House, and a practical statement of "our next duty." Much good may be done by a brief account like this of the work and sacrifice of one good man, and we cordially recommend it to all who appreciate heroism in whatever field displayed.

**A More Excellent Way.** †

This is a disappointing book, mainly because, in attempting much, the authoress has gone beyond her powers and accomplished little. The seed that in Mrs. Humphrey Ward found such good soil and produced "Robert Elsmere" seems here

* "Father Damien: a Journey from Cashmere to His Home in Hawaii." By Edward Clifford. (Macmillan and Co.)

† "A More Excellent Way." By Constance Howell. (Swan Sonnenschein, Lowrey, and Co.)

to have fallen by the wayside, and developed into a sickly plant that is little likely to thrive. For, truth to tell, "A More Excellent Way" is very weak. Apparently dissatisfied with nineteenth century Christianity and our latter-day social conditions, Mrs. Howell has, like many another enthusiast, attempted to draw for us architect's plans of a different society, and has failed to give them a semblance of possibility. It seems as if material had been collected for two separate books, and as if, when both had been partly written, for some reason, best known to the author, they had been converted into one. The dove-tailing is bad, and altogether the work lacks finish. "Mrs. Hathaway," in the words of one of the characters, "is a perfect lady, and her son is most intelligent and nice." That is the hero's failing. Rich, good-looking, gentlemanly, an Atheist, and a Socialist, he apparently possesses all that should constitute an ideal hero in these novels with something to preach, and yet he only succeeds in being nice. "Nice" men should be impossible, like Sir Arthur Helps' "tolerable eggs." The minor characters are unobtrusive if somewhat commonplace, but we think Otho Hathaway should be congratulated on his escape from marriage with Miss Evangeline Champneys. If people with religious and socialistic theories to advocate would publish them in the form of sermons or pamphlets, fewer poor novels would be thrust upon an innocent and deserving public.

### Chats at St. Ampelio.*

Mankind owes much more gratitude than it usually expresses to those who have written books that serve to occupy the odd minutes of life. The great authors demand our leisure hours; we take them a canto at a time. The minor poets and essayists charm the spare five minutes. We may read a stanza or two of Herrick and Béranger, or a chapter of Montaigne, and put the book down without feeling that we have done the author an injustice. Such gratitude is due to Dr. Goodchild for his "Chats at St. Ampelio." From dedication to *finis* his book is entirely charming, freshly written, full of quaint conceits and dainty suggestions. His humour is delightfully spontaneous and refreshing, and while he may be held quite innocent of plagiarism, there are many touches which recall blended recollections of Wilson, Lamb, and Helps. This is high praise, but we think well merited. It is impossible to select quotations at all representative of the tone of the book, but perhaps the chapter on "Sorrows and Symbols" is one of the most delightful. This is one of those books that we not only advise people to read, but to possess, and it will serve to while away, with pleasure and profit, many a spare quarter of an hour, and many a sleepless night.

### Ralph Ellison's Opportunity.†

This volume contains two stories of which the first, "East and West," is the longer and better. Mr. Keith has a vigorous touch, and considerable skill in delineation of character. In "East and West" he has chosen the somewhat well-worn theme of a young poet's struggles in the asphyxiating atmosphere of Bethnal Green, but he has kept his individuality, despite a certain tendency towards Besant's treatment and style. Alicia Frankland, the heroine, is excellent, original, amusing, and womanly. The dialogue is crisp, and the story well put together. "Ralph Ellison's Opportunity" does not show quite so much ability perhaps, but it is wholesome and thoroughly readable, and the volume, which is well got up and contains several very fair illustrations, will form a sensible present for boys and girls alike.

### Subjects of Social Welfare.‡

Speeches and addresses are at first sight likely to be considered as of only ephemeral interest, devoted as they are to special subjects and to special audiences. Presumably, they are intended to enlighten a particular section of the great public on some particular topic of temporary interest. Magazine articles, again, are of somewhat similar nature, with this difference, that the section of the public to which they are addressed is wider and often more intelligent. But the underlying notion that they are ephemeral is more or less true of them all, and generally speaking they are relegated to oblivion and lose their individual reputation after contributing their quota to the making of history. There are, however, some subjects of chronic interest, which periodically become prominent and of ephemeral importance. As instances we may quote vaccination, vivisection, disposal of the dead, protection, marriage with a deceased wife's sister, and so on. Without being in any sense questions for political parties, they are from their very nature perpetually recurring and perpetually being debated on the platform and in the Press. It is extremely unlikely that they will ever be finally disposed of, and hence it is well that the spoken and written opinions of individuals, whose authority and right to a hearing are undisputed, should be rescued from the files of newspapers and magazines and reprinted in book form, and so made more easily accessible for future reference. Sir Lyon Playfair has thus dealt with several of his contributions to these periodical discussions, and has republished them under the apt and comprehensive title, "Subjects of Social Welfare." The volume is divided into three sections, treating respectively of Public Health, Industrial Wealth, and National Education. It includes addresses to the Social Science Association, the Royal Institution, Manchester, the National Liberal Club, speeches delivered in Parliament and to his constituents at Leeds, and contributions to various magazines. By laymen as well as by professional political economists this book is likely to be cordially received. Sir Lyon Playfair's language is remarkably plain and free from technical mannerisms, his arguments are lucid, and his facts well marshalled. He wastes no time in useless verbiage, but goes straight to the point he intends to demonstrate or controvert, and with all this never sins by being dull or tedious. Owing probably to the fact that the volume is a collection of speeches and articles produced on separate occasions, and not a book written, so to speak, at one time, there is a certain amount of over-lapping and reiteration. This is especially noticeable in the paper on the "Displacement of Labour by Modern Inventions," published originally in the *Contemporary Review*, and an address on "Industrial Competition and Commercial Freedom," delivered to the National Liberal Club in 1888, and which in this volume come immediately next to one another. The speech in defence of vivisection is a concise résumé of the principal arguments for and against, ably elucidated, and, as we think, convincingly established. The reply to the resolution moved by Mr. Taylor in 1883 on behalf of the Anti-vaccination Association is of special interest again now, in the light of the further development of the question as set forth in Mr. Crookshank's recent book. Perhaps the most able portion of the volume is that devoted to industrial wealth. The four articles on "Depression of Agriculture and Fair Trade," "The Displacement of Labour by Modern Inventions," "Industrial Competition and Commercial Freedom," and "The Effect of Protection on Wages" are particularly powerful, although, as we have said, it is here that the fault of overlapping is most apparent —if under the circumstances it be a fault. The third, and last section, treating of national education, is of high interest and of increasing importance in these days when competition is passing from material to mental and intellectual regions. Sir Lyon Playfair's book will form a useful addition to the library of everyone who takes a practical interest in the social problems of the day. He propounds his views simply, and if we disagree with some of his conclusions we must admire his methods of reasoning. Convenient in size, and agreeably printed, "Subjects of Social Welfare" contains a mine of information that many will find pleasure in exploring.

* "Chats at St. Ampelio." By John A. Goodchild. (Kegan Paul, Trench and Co.)

† "Ralph Ellison's Opportunity; and East and West." By Leslie Keith. (The Religious Tract Society.)

‡ "Subjects of Social Welfare." By the Right Hon. Sir Lyon Playfair, K.C.B., M.P., LL.D., F.R.S. (Cassell and Co.)

